# The Temporal Distribution and Microbial Spectrum in Infectious Keratitis: A Comprehensive Single-Center Study

**DOI:** 10.3390/jcm14051613

**Published:** 2025-02-27

**Authors:** Naoyuki Yamada, Nanako Iwamoto, Ayano Sakuma, Junki Sunada, Ren Aoki, Masanori Mikuni, Fumiaki Higashijima, Takuya Yoshimoto, Yukiko Morita, Kazuhiro Kimura

**Affiliations:** 1 Department of Ophthalmology, School of Medicine, Yamaguchi University, Ube 755-8505, Japan; iwamo@yamaguchi-u.ac.jp (N.I.); sakuma-a@yamaguchi-u.ac.jp (A.S.); sunada@yamaguchi-u.ac.jp (J.S.); renaoki@yamaguchi-u.ac.jp (R.A.); mikuni89@yamaguchi-u.ac.jp (M.M.); f.higashijima@gmail.com (F.H.); azyumi08@gmail.com (T.Y.); k.kimura@yamaguchi-u.ac.jp (K.K.); 2 Obata Eye Clinic, Iwakuni 741-0062, Japan; ymorita@obataganka.jp

**Keywords:** infectious keratitis, seasonality, *Pseudomonas aeruginosa*, Acanthamoeba, HSV

## Abstract

**Purpose:** To elucidate pathogen-specific seasonal patterns in infectious keratitis through a comprehensive long-term analysis of microbiologically proven cases in a Japanese tertiary care center. **Methods:** This retrospective study analyzed 500 consecutive cases of culture-proven infectious keratitis diagnosed and treated by corneal specialists at Yamaguchi University Hospital between 2009 and 2021. Seasonal distribution patterns were analyzed for each pathogen category and specific microorganisms. **Results:** Among the 500 cases, bacteria were identified in 249 eyes (49.8%), viruses in 173 eyes (34.6%), fungi in 51 eyes (10.2%), and Acanthamoeba in 27 eyes (5.4%). The top 10 causative microorganisms constituted 80.4% of all cases. Distinct seasonal patterns emerged: bacterial keratitis peaked during winter months (October–March), *Pseudomonas aeruginosa* infections clustered in late summer (August–September), Acanthamoeba keratitis showed summer predominance (June–August), and HSV keratitis was most frequent in winter to spring (January–May). Overall incidence peaked from January to March and reached its nadir in June. **Conclusions:** This long-term study revealed distinct seasonal patterns for specific pathogens causing infectious keratitis in Japan. The findings suggest that geographical location and climate may influence the temporal distribution of corneal infections. These pathogen-specific seasonal trends could aid in the preliminary diagnosis and empirical treatment of infectious keratitis.

## 1. Introduction

The cornea is a transparent avascular tissue located at the surface of the eyeball. It acts as a structural barrier and protects the eye. The cornea is constantly exposed to external stresses, such as trauma and infection. Since the cornea is located along the optical axis, corneal opacity directly leads to loss of vision. Infections caused by pathogenic microorganisms in the cornea can be defined as infectious keratitis [1,2]. In general, when pathogenic microorganisms establish an infection in the cornea, corneal epithelial defects occur, which progress to corneal ulceration. Corneal perforation may occur in some cases if the corneal stroma melts. Corneal perforation makes it difficult to maintain the shape of the eye. Endophthalmitis can occur if the corneal infection spreads to the anterior chamber, vitreous, and retina. Consequently, the risk of blindness is high in such cases. If severe inflammation or prolonged healing occurs, the cornea may be scarred and develop an abnormal shape, resulting in reduced transparency and visual impairment [3,4]. Thus, infective keratitis is a serious ocular disease that can lead to corneal melting and perforation in severe cases, resulting in blindness. Diverse microorganisms, including bacteria, fungi, viruses, and amoebas, can cause infectious keratitis. For example, in Japan, the “Guidelines for Clinical Management of Infectious Keratitis (2nd edition)”, formulated by the Japanese Ophthalmological Society, lists 28 causative microorganisms for infectious keratitis [5]. The identification of the exact microorganism that causes infectious keratitis among the 28 species is an arduous task. Several of these 28 species are the common causative microorganisms of infectious keratitis in countries other than Japan. The accurate identification of the causative microorganism is required for selecting the drug to be administered for the treatment of infectious diseases [6]. However, infectious keratitis is an infection of the cornea, a relatively small organ measuring approximately 11 mm in diameter. The amount of sample collected by abrasion of the infected foci in the cornea for the identification of the causative microorganisms is very small; therefore, the identification rate is never satisfactory [7]. Furthermore, different causative microorganisms may present with similar corneal findings, whereas the same causative microorganism may present with different corneal findings in different cases. In particular, differentiating between bacterial and fungal keratitis on the basis of corneal findings alone is an arduous task. We previously reported that the microorganism causing infectious keratitis was identified in only 72% cases using culture tests or microscopy [8]. Even with the latest artificial intelligence (AI) technology, the diagnosis of infectious keratitis is limited to differentiating between bacteria, fungi, herpes, and amoebae [9]. Therefore, in the context of infectious diseases, it is advantageous to narrow down the candidate causative microorganisms to select the drug to be administered. Seasonality can be defined as the presence of variations that occur at specific regular intervals of less than a year. Previous studies have reported that pathogenic microorganisms exhibit distinct seasonal patterns [10]. For example, influenza viruses are prevalent mainly during winter, while other pathogens show different seasonal preferences [11]. Several studies from various countries have reported seasonal trends in infectious keratitis [12,13,14]. Although there have been reports on the seasonality of infectious keratitis from Europe, North America, South America, South Asia, and Oceania, there have been no reports from East Asia, including Japan, as far as we know. Furthermore, most of these studies on infectious keratitis-causing microorganisms have been limited to bacteria, fungi, and Acanthamoeba, with very few on the very frequently involved viruses, namely HSV, VZV, and CMV. Viruses are also very common causative microorganisms of infectious keratitis. That is why we decided to include viruses in this study. However, comprehensive analyses of pathogen-specific seasonal patterns in infectious keratitis remain limited. The development of infectious keratitis is influenced by multiple factors, including the virulence of pathogens, host immunity, and environmental conditions such as temperature and humidity. Understanding the seasonal patterns of specific pathogens could provide valuable insights for clinical diagnosis and treatment strategies. Therefore, we investigated the temporal distribution patterns of infectious keratitis by analyzing the relationships between specific pathogens and their seasonal occurrence.

## 2. Materials and Methods

Data were retrospectively collected from the medical records of the study participants and examined. This study was approved by the institutional review board of Yamaguchi University. The School of Medicine complied with the tenets of the Declaration of Helsinki while performing this study. The institutional review board approved the use of opt-out consent method. Informed consent was waived by the institutional review board of Yamaguchi University Hospital. This study was approved by the Ethical Review Board of the Research Ethics Committee (REC) of Yamaguchi University Hospital (2020-215).

This retrospective study included 500 consecutive patients diagnosed with infectious keratitis by a single corneal specialist between January 2009 and January 2021 at the Department of Ophthalmology, Yamaguchi University Hospital, Yamaguchi, Japan.

Since some cases were bilateral or caused repeated infections in the same eye, there were a total of 500 treatment episodes in 379 patients with 391 eyes. The mean age (±standard deviation) of the study population was 63 ± 22 years, 270:230 for males and females, and 242:258 for the right and left eye, respectively. Of the 500 cases of infectious keratitis, 148 (29.6%) occurred in patients with a history of keratoplasty. In principle, the causative microorganisms of infectious keratitis were detected at the time of the first visit using microbiological culture tests of samples obtained from corneal abrasion, discharge, and Contact Lens Preservation Solution, or by Micro Trak (virus-specific antigen test) or anterior chamber aqueous PCR if viral infection was suspected. However, HSV was defined when clear dendritic or geographic keratitis was confirmed using photographs from a previous physician, whereas pseudodendritic keratitis during or immediately after the onset of herpes zoster was defined as VZV. HSV and VZV cases included not only the epithelial type but also the stromal and endothelial types, which are mainly caused by immune reactions, as well as cases in which these types occurred simultaneously. Once keratitis caused by HSV, VZV, or CMV was diagnosed, subsequent recurrences were considered to be caused by the same virus if the corneal findings were consistent.

The month of onset of infectious keratitis (mainly the date of examination when the causative microorganisms were identified) was retrospectively examined.

If several causative microorganisms were detected in the same eye on the same day, one was chosen based on the corneal findings. For example, when *Pseudomonas aeruginosa* and Enterobacter cloacae complex were detected, infection by *Pseudomonas aeruginosa* was assumed if corneal findings such as ring-shaped abscesses were evident.

The monthly incidence patterns of the causative microorganisms of infectious keratitis were created using PowerPoint (Microsoft). For the monthly incidence patterns of the causative microorganisms of infectious keratitis (absolute value display), the color of the cells in December for Others, with the highest number of cases (15) by month for each causative microorganism, is shown as “black, 0% transparency” (i.e., black), and the color of the cells in months with zero cases is shown as “black, 100% transparency” (i.e., white). For monthly incidence patterns of causative microorganisms of infectious keratitis (monthly relative value display), the cells of the causative microorganisms with the highest number of cases in each month are colored “black, 0% transparency” (i.e., black), and the cells of months with zero cases are colored “black, 100% transparency” (i.e., white).

## 3. Results

**Figure 1:**
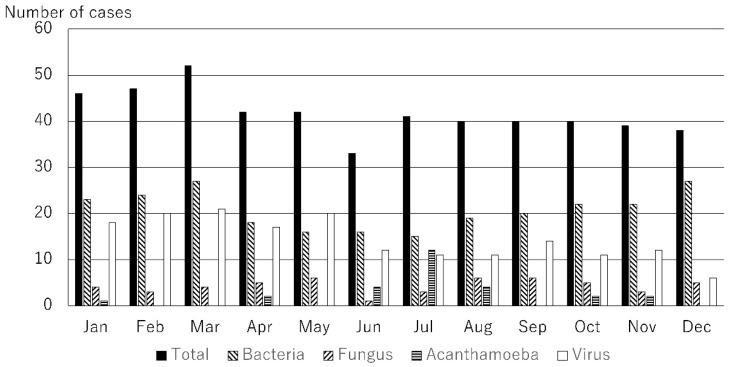
Number of cases of infectious keratitis by month caused by total, bacteria, fungi, Acanthamoeba, and viruses.

**Figure 2:**
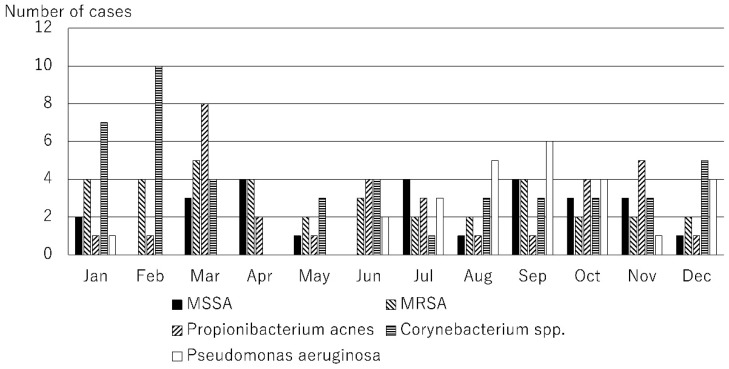
The number of infectious keratitis cases per month for MSSA, MRSA, *Propionibacterium acnes*, *Corynebacterium* spp., and *Pseudomonas aeruginosa*. These microbes were among the top 10 causative microorganisms.

**Figure 3:**
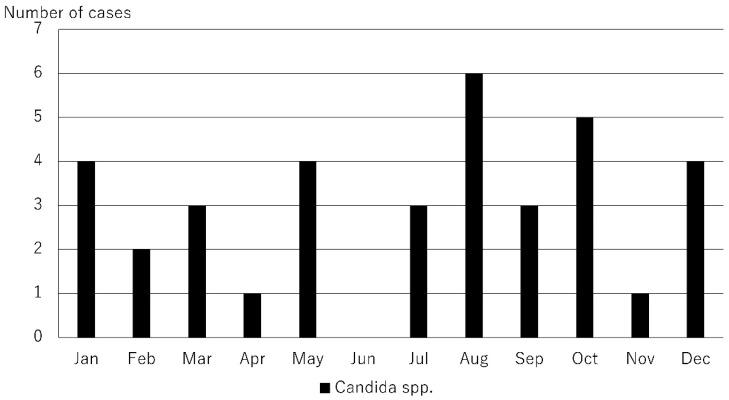
Number of cases of Candida keratitis by month. Number of cases of infectious keratitis per month caused by *Candida* spp. Candida was included in the top 10 causative microorganisms.

**Figure 4:**
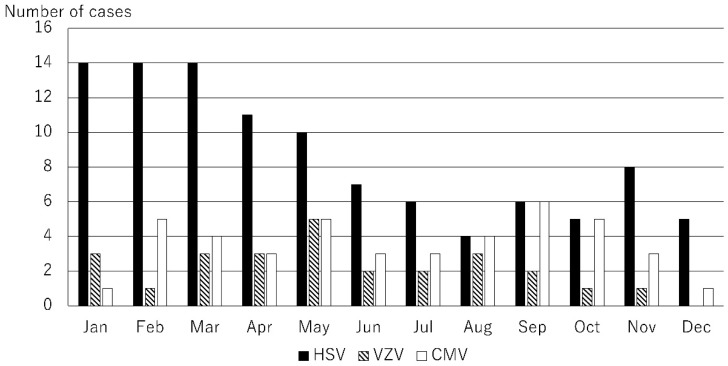
The number of cases of viral keratitis by month. The number of infectious keratitis cases per month caused by HSV, VZV, and CMV. These viruses were among the top 10 causative microorganisms.

**Figure 5:**
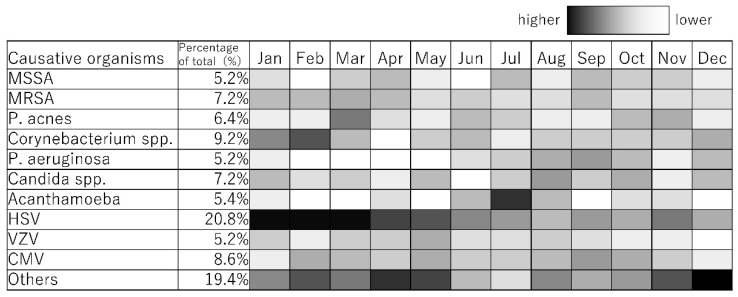
Monthly incidence patterns of causative microorganisms of infectious keratitis (absolute value display).

**Figure 6:**
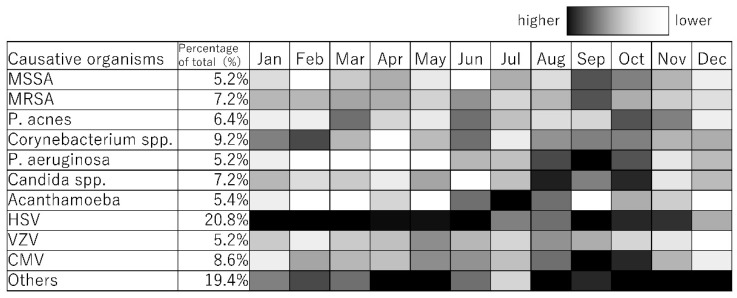
Monthly incidence patterns of causative microorganisms of infectious keratitis (monthly relative value display).

The causative microorganisms included 249 bacteria (49.8%), 51 fungi (10.2%), 27 Acanthamoeba (5.4%), and 173 viruses (34.6%). A total of 78 causative microorganisms were detected using microbiological culture tests, Micro Trak, or anterior chamber aqueous polymerase chain reaction (PCR). The number of cases of infectious keratitis caused by bacteria, fungi, Acanthamoeba, and viruses is shown in Figure 1. The number of cases of infectious keratitis was higher in January (46), February (47), and March (52), and lower in June (33). The number of bacterial infections was highest in October (22), November (22), December (27), January (23), February (24), and March (27). Fungal infections ranged from one to six cases per month with no clear seasonality. The number of cases caused by Acanthamoeba was higher during the summer months of June (4), July (12), and August (4). The number of viral infections was highest in January (18), February (20), March (21), April (17), and May (20). This analysis was based on the data for the top 10 causative organisms, which accounted for 80.4% (402/500) of all cases (Table 1). Causative microorganisms ranked ≤11 were classified as others. The top 10 species causing infectious keratitis are listed in Table 1. All these causative microorganisms were among the 28 causative agents listed in the Guidelines for Clinical Management of Infectious Keratitis. Of these, the number of cases caused by bacterial species, such as methicillin-sensitive Staphylococcus aureus (MSSA), methicillin-resistant Staphylococcus aureus (MRSA), *Propionibacterium acnes*, *Corynebacterium* spp., and *Pseudomonas aeruginosa* are shown in Figure 2; those caused by fungal species, such as *Candida* spp., are shown in Figure 3; those by Acanthamoeba are shown in Figure 1; and those by viruses, such as herpes simplex virus (HSV), varicella zoster virus (VZV), and cytomegalovirus (CMV), are shown in Figure 4. MSSA was multimodal and showed no clear seasonality in causing infectious keratitis. The prevalence of MRSA-induced infectious keratitis was higher in January (4), February (4), March (5), April (4), and September (4). The rate of *Propionibacterium acnes* being the cause of infection was higher in March (8), and *Corynebacterium* spp. was prevalent from January (7) to February (10). *Pseudomonas aeruginosa*-induced infectious keratitis was higher from August (5) to September (6) (Figure 2). *Candida* spp. was multimodal and showed no clear seasonality (Figure 3) in causing infection. HSV-induced infectious keratitis was more common in January (14), February (14), and March (14), whereas VZV and CMV were multimodal and showed no clear seasonality (Figure 4). Based on the results of this study, we found distinct monthly incidence patterns for the microorganisms causing infectious keratitis (absolute values displayed in Figure 5, monthly relative values displayed in Figure 6).

## 4. Discussion

In this study, we identified the pathogen-specific seasonal patterns of infectious keratitis through a comprehensive analysis of microbiologically proven cases. The incidence of infectious keratitis was highest from January to March and lowest in June. Distinct seasonal patterns were observed: bacterial infections predominated between October and March, *Pseudomonas aeruginosa* infections clustered in August to September, Acanthamoeba infections were common in summer (June to August), and HSV infections peaked between January and March. Fungal infections and some viral pathogens (VZV and CMV) showed no clear seasonality. Several studies from outside Japan have discussed seasonality in infectious keratitis [13,15]. A study from Nottingham, England, indicated that Gram-positive rods more commonly caused infectious keratitis during the summer season [16]. On the contrary, in this study, *Propionibacterium acnes*-induced infection was found to be more common in March, and *Corynebacterium* spp.-induced infection in January and February. The aforementioned study also reported that *Pseudomonas aeruginosa* more commonly caused infection in the summer, which is in line with the present study. A study from Manchester, England, reported that Gram-negative bacteria-induced infections were more common during the summer and fall seasons [17]. A report from Toronto, Canada, showed that Acanthamoeba-induced infectious keratitis was common during the summer season, which is consistent with the present study [18]. Studies from Brisbane and Sydney, Australia, demonstrated that *Pseudomonas aeruginosa* more commonly caused infections during summer, which is consistent with the results of the present study [12,19]. A report from southeast India showed that *Pseudomonas aeruginosa*-induced infectious keratitis was common from July to December, which is partially consistent with the results of the present study [13]. Since southeast India belongs to the tropics, with few seasonal changes and constantly high temperatures, it is thought that the crisis of *Pseudomonas aeruginosa* was high not only during a particular season, but over multiple seasons. A study from Brazil showed that bacterial infections were common from fall to winter, which is consistent with the results of the present study [15].

Yamaguchi Prefecture, where this study was conducted, is located at 34° N, 131° E and is the westernmost point of Japan’s Honshu Island. Yamaguchi Prefecture is surrounded by sea in three directions, and the four seasons are rich in variation. It is difficult to make comparisons with tropical countries such as India and Brazil because, unlike in Japan, there is little distinction among the four seasons. However, reports from the United Kingdom in the Northern Hemisphere and Australia in the Southern Hemisphere, both of which belong to temperate zones like Yamaguchi, are worth comparing. Therefore, it can be elicited that the increased incidence of infectious keratitis caused by *Pseudomonas aeruginosa* and Acanthamoeba, as demonstrated in this study, also occurs worldwide during summer. The United Kingdom and Australia are developed countries, similar to Japan, and their sanitary conditions are considered to be relatively good.

Besides infectious keratitis, it has been reported that there is a positive correlation between the incidence of urinary tract infections caused by *Pseudomonas aeruginosa* and either temperature or precipitation [20]. Therefore, we hypothesized that *Pseudomonas aeruginosa* infections were more common in Japan during the summer season, when temperatures were relatively high and precipitation was abundant.

To the best of our knowledge, there are no previous reports on the seasonality of viral keratitis. A very high proportion of infectious keratitis cases were caused by viruses; therefore, they were included in this study. We found that HSV-induced keratitis was common between January and May. Furthermore, this study is the first to have determined the monthly frequencies of the 10 most frequent causative microorganisms of infectious keratitis (Figure 5 and Figure 6). Since this study was conducted at a university hospital, the severity and timing of disease onset may differ slightly from cases in the clinic. It has been observed that mild cases are cured completely in the clinics, while severe cases are transferred to university hospitals. Therefore, the data included in this study may have been shifted to the slightly more severe side of infectious keratitis. In addition, the time of onset of keratitis may have been delayed by a few days because this study was conducted at a university hospital rather than at clinics. Since this study involved monthly evaluations, it was considered that this had no significant effect. The monthly incidence patterns presented in both absolute values (Figure 5) and relative proportions (Figure 6) provide complementary insights for clinical practice. The relative monthly display is particularly useful for clinical decision-making. For example, if a patient with infectious keratitis is examined in July, the higher prevalence of Acanthamoeba during this period should be considered alongside clinical findings and laboratory results. Understanding these pathogen-specific seasonal patterns could assist clinicians in making preliminary diagnoses and selecting appropriate empirical treatments.

## 5. Conclusions

This long-term study revealed distinct seasonal patterns for specific pathogens causing infectious keratitis in Japan. The findings suggest that geographical location and climate may influence the temporal distribution of corneal infections. These pathogen-specific seasonal trends could aid in the preliminary diagnosis and empirical treatment of infectious keratitis. While accurate differentiation between infectious and non-infectious conditions remains crucial, these monthly incidence patterns may serve as a valuable additional tool for ophthalmologists in diagnosing infectious keratitis. This study is limited by the experience of a single corneal specialist at a single institution. Future national and international studies are required to prove the generalizability of our results at the domestic and universal levels.

## Figures and Tables

**Table 1:** Top ten causative microorganisms.

Causative Organisms	Number of Cases
HSV	104
*Corynebacterium* spp.	46
CMV	43
MRSA	36
*Candida* spp.	36
*Propionibacterium acnes*	32
Acanthamoeba	27
MSSA	26
*Pseudomonas aeruginosa*	26
VZV	26
Total	402 (80.4%)

## Data Availability

The datasets used and/or analyzed during the current study are available from the corresponding author on reasonable request.

## References

[1] Sean L., Edelstein P.W., Andrew J.W., Huang A., Krachmer J.H., Mannis M.J., Holland E.J. (2011). Bacterial Keratitis. Cornea.

[2] Stapleton F. (2023). The epidemiology of infectious keratitis. Ocul. Surf..

[3] Khor W.B., Prajna V.N., Garg P., Mehta J.S., Xie L., Liu Z., Padilla M.D.B., Joo C.K., Inoue Y., Goseyarakwong P. (2018). The Asia Cornea Society Infectious Keratitis Study: A Prospective Multicenter Study of Infectious Keratitis in Asia. Am. J. Ophthalmol..

[4] Menda S.A., Das M., Panigrahi A., Prajna N.V., Acharya N.R., Lietman T.M., McLeod S.D., Keenan J.D. (2020). Association of Postfungal Keratitis Corneal Scar Features with Visual Acuity. JAMA Ophthalmol..

[5] Zasshi N.G. (2013). Guidelines for the clinical management of infectious keratitis. Nippon Ganka Gakkai Zasshi.

[6] Intra J., Sala M.R., Falbo R., Cappellini F., Brambilla P. (2016). Reducing time to identification of aerobic bacteria and fastidious micro-organisms in positive blood cultures. Lett. Appl. Microbiol..

[7] Alkatan H.M., Al-Essa R.S. (2019). Challenges in the diagnosis of microbial keratitis: A detailed review with update and general guidelines. Saudi J. Ophthalmol..

[8] Harada D., Chikama T., Yamada N., Nomi N., Kawamoto K., Nishida T. (2009). Value of microscopic examination of smear in corneal infections. Rinsho Ganka.

[9] Koyama A., Miyazaki D., Nakagawa Y., Ayatsuka Y., Miyake H., Ehara F., Sasaki S.I., Shimizu Y., Inoue Y. (2021). Determination of probability of causative pathogen in infectious keratitis using deep learning algorithm of slit-lamp images. Sci. Rep..

[10] Wang J., Huang J.J., Lynch I. (2022). Seasonal and short-term variations of bacteria and pathogenic bacteria on road deposited sediments. Environ. Res..

[11] Neumann G., Kawaoka Y. (2022). Seasonality of influenza and other respiratory viruses. EMBO Mol. Med..

[12] Khoo P., Cabrera-Aguas M.P., Nguyen V., Lahra M.M., Watson S.L. (2020). Microbial keratitis in Sydney, Australia: Risk factors, patient outcomes, and seasonal variation. Graefes Arch. Clin. Exp. Ophthalmol..

[13] Lin C.C., Lalitha P., Srinivasan M., Prajna N.V., McLeod S.D., Acharya N.R., Lietman T.M., Porco T.C. (2012). Seasonal trends of microbial keratitis in South India. Cornea.

[14] Gorski M., Genis A., Yushvayev S., Awwad A., Lazzaro D.R. (2016). Seasonal Variation in the Presentation of Infectious Keratitis. Eye Contact Lens.

[15] Marujo F.I., Hirai F.E., Yu M.C., Hofling-Lima A.L., Freitas D., Sato E.H. (2013). Distribution of infectious keratitis in a tertiary hospital in Brazil. Arq. Bras. Oftalmol..

[16] Ting D.S.J., Ho C.S., Cairns J., Gopal B.P., Elsahn A., Al-Aqaba M., Boswell T., Said D.G., Dua H.S. (2021). Seasonal patterns of incidence, demographic factors and microbiological profiles of infectious keratitis: The Nottingham Infectious Keratitis Study. Eye.

[17] Walkden A., Fullwood C., Tan S.Z., Au L., Armstrong M., Brahma A.K., Chidambaram J.D., Carley F. (2018). Association Between Season, Temperature and Causative Organism in Microbial Keratitis in the UK. Cornea.

[18] McAllum P., Bahar I., Kaiserman I., Srinivasan S., Slomovic A., Rootman D. (2009). Temporal and seasonal trends in *Acanthamoeba keratitis*. Cornea.

[19] Green M., Apel A., Stapleton F. (2008). A longitudinal study of trends in keratitis in Australia. Cornea.

[20] Ramos G.P., Rocha J.L., Tuon F.F. (2013). Seasonal humidity may influence Pseudomonas aeruginosa hospital-acquired infection rates. Int. J. Infect. Dis..

